# A Steady-State Kalman Predictor-Based Filtering Strategy for Non-Overlapping Sub-Band Spectral Estimation

**DOI:** 10.3390/s150100110

**Published:** 2014-12-24

**Authors:** Zenghui Li, Bin Xu, Jian Yang, Jianshe Song

**Affiliations:** 1 Department of Electronic Engineering, Tsinghua University, Beijing 100084, China; E-Mails: lizenghui11@mails.tsinghua.edu.cn (Z.L.); b-xu11@mails.tsinghua.edu.cn (B.X.); 2 Xi'an Research Institute of Hi-Technology, Xi'an 710025, China; E-Mail: Songjianshe09@126.com

**Keywords:** AR model, equiripple FIR filter, linear prediction, spectral estimation, spectral overlap, sub-band decomposition

## Abstract

This paper focuses on suppressing spectral overlap for sub-band spectral estimation, with which we can greatly decrease the computational complexity of existing spectral estimation algorithms, such as nonlinear least squares spectral analysis and non-quadratic regularized sparse representation. Firstly, our study shows that the nominal ability of the high-order analysis filter to suppress spectral overlap is greatly weakened when filtering a finite-length sequence, because many meaningless zeros are used as samples in convolution operations. Next, an extrapolation-based filtering strategy is proposed to produce a series of estimates as the substitutions of the zeros and to recover the suppression ability. Meanwhile, a steady-state Kalman predictor is applied to perform a linearly-optimal extrapolation. Finally, several typical methods for spectral analysis are applied to demonstrate the effectiveness of the proposed strategy.

## 1. Introduction

As one of the most important tools, spectral estimation [1] has been extensively applied in radar, sonar and control systems, in the economics, meteorology and astronomy fields, speech, audio, seismic and biomedical signal processing, and so on. In particular, sparse representation [2–4] opens an exciting new vision for spectral analysis. However, such methods are usually accompanied by high computational complexity, which makes their availability somewhat limited.

Sub-band decomposition-based spectral estimation (SDSE) [5] is an important research direction in spectral estimation, because it has several advantageous features, e.g., computational complexity decrease, model order reduction, spectral density whiteness, reduction of linear prediction error for autoregressive (AR) estimation and the increment of both frequency spacing and local signal-to-noise ratio (SNR) [6]. These features have been theoretically demonstrated under the hypothesis of the ideal infinitely-sharp bandpass filter bank [7]. Subsequent studies [8–10] indicate that these benefits aid complex frequency estimation in sub-bands, thereby enabling better estimation performance than that achieved in full-band. In addition, the computational complexity of most algorithms for spectral analysis has a superlinear relationship with the data size, and sub-band decomposition can considerably speed up these algorithms. Independently handling each sub-band enables parallel processing, which can further improve the computational efficiency. Both advantages are crucial for reducing the computational burden, especially when analyzing multi-dimensional big data, such as polarimetric and/or interferometric synthetic aperture radar images of large scenes.

Unfortunately, the ideal infinitely-sharp bandpass filter cannot be physically realized, and non-ideal (realizable) filters introduce energy leakage and/or frequency aliasing phenomena [11]. Due to these non-ideal frequency characteristics of analysis filters, spectral overlap between any two contiguous sub-bands occurs during the sub-band decomposition. Then, the performance of SDSE severely degrades.

In the relevant literature, several methods have been proposed to mitigate spectral overlap. We classify these methods into three categories. The first category is defined as ideal frequency domain filtering with a strict box-like spectrum, such as “ideal” Hilbert transform-based half-band filters [9] and harmonic wavelet transform-based filters [12,13]. Theoretically, sub-band decomposition with these filters is immune to spectral overlap. However, discrete Fourier transform will inevitably induce spectral energy leakage, which can likewise distort sub-band decomposition. The second category is known as convolution filtering with wavelet packet filters [8], Kaiser window-based prototype cosine modulated filters, discrete cosine transform (DCT) IV filters [10] and Comb filters [6,14]. It seems that increasing the filter order can improve the filtering performance and also the spectral overlap suppression capability. However, in the context of involving a finite-length sequence and performing convolution filtering, the nominal improvement of performance will lead to spectral energy leakage and inferior filtering accuracy [10]. Considering the compromise between suppressing spectral overlap and reducing spectral energy leakage, we have to restrict the filter order. The third category is frequency-selective filtering, and a representative method is SELF-SVD (singular value decomposition-based method in a selected frequency band) [15]. Essentially, SELF-SVD attempts to attenuate the interferences of the out-of-band components by the post-multiplication with an orthogonal projection matrix. Unfortunately, the attenuation is often insufficient when the out-of-band components are much stronger than the in-band components or the SNR is relatively low. In this case, the estimation of the in-band frequencies is seriously affected.

In this paper, a new filtering strategy is proposed to suppress spectral overlap for sub-band spectral estimation. First, we discuss the formation mechanism of spectral overlap. Nominally, a high-order finite impulse response (FIR) filter usually has a powerful ability in spectral overlap suppression. However, once we perform such a filter on a finite-length sequence with the convolution operation, the non-given samples at the forward and backward sampling periods of the sequence are assumed to be zeros. A certain filtering error therefore occurs and conversely disrupts the decomposed sub-bands. As a result, sub-band spectral analysis severely suffers from the mutual overlap of adjacent sub-band spectra. Second, we propose a filtering strategy to eliminate the filtering error and recover the suppression ability. This strategy intuitively takes the place of the artificial zeros with some extrapolated samples. Toward the problem of data extrapolation, many algorithms have been proposed based on various theories, such as linear prediction [16], Gerchberg–Papoulis [17], Slepian series [18], linear canonical transform [19] and sparse representation [20]. To establish an efficient method for the extrapolation in context and to evaluate the effectiveness of the proposed strategy, we preliminarily develop a linearly-optimal extrapolation based on the classical AR model identification and the Kalman prediction [21–23]. Third, we derive the formulas to estimate the residual filtering error and adapt two common information criteria with adaptive penalty terms for AR order determination. Moreover, equiripple FIR filters are applied as analysis filters in coordination with the proposed filtering, because of their advantageous features [11]. Finally, the entire algorithm and the computational complexity are summarized. Some details, such as the sub-band spectrum mosaicking procedure and parameter selection, are discussed in practice.

The remainder of the paper is organized as follows. In Section 2, the formation mechanism of spectral overlap is discussed. Based on this, a steady-state Kalman predictor-based filtering strategy is developed to suppress the overlapped spectra. In Section 3, the proposed filtering strategy is discussed for SDSE. In Section 4, experimental results with several typical algorithms for spectral analysis demonstrate the effectiveness of the proposed strategy. Finally, Section 5 concludes this paper.

## 2. Signal Filtering Based on AR Model Identification and Kalman Prediction

This section focuses on signal filtering. To reduce the filtering error induced by convolution filtering, we propose an extrapolation-based filtering strategy and apply a steady-state Kalman predictor for extrapolation. Two criteria with adaptive penalty terms for order determination are developed based on the estimation of the residual filtering error.

### 2.1. Problem Statement of Signal Filtering

FIR filters are typical linear time-invariant (LTI) systems. According to the linear system theory, the filter can be mathematically expressed as the convolution of its impulse response with the input. Suppose that {*x_n_*} is an input sequence and {*h_n_*} is the impulse response of a causal FIR filter; the filtered sequence {*y_n_*} can be derived as [11]:

(1) $$y_{n}=h_{n}*x_{n}=\sum_{k=0}^{N_{f}-1}h_{k}x_{n-k}$$

where * denotes the convolution operator and *N_f_* is the filter length (*i.e.*, the length of the impulse response; the relationship between *N_f_* and the filter order *N_o_* can be written as *N_f_* = *N_o_* + 1). Alternatively, taking discrete-time Fourier transforms (DTFT), we can represent Equation (1) in the frequency domain as:

(2) $$Y\left(e^{j\omega }\right)=H\left(e^{j\omega }\right)X\left(e^{j\omega }\right)$$

In addition, the filtered sequence length *L*, the input sequence length *N* and the filter length *N_f_* satisfy:

(3) $$L=N+N_{f}-1=N+N_{o}$$

Theoretically, given a large enough stop-band attenuation, spectral overlap can be thoroughly suppressed. Moreover, the spectral estimation error in sub-bands can be neglected, as long as the width of the transition band and the ripple of the passband are sufficiently small. Nonetheless, the pursuit of excellent filtering performance substantially increases both the filter order and the length of the filtered sequence (refer to Equation (3)). Such a high order is more likely to create error in part or even all of the filtered samples. This result is contrary to our original objective, and the resultant filtering quality is undesirable.

From the perspective of a discrete-time system, the output sequence of the convolution operation is equivalent to the zero-state response of the filter system, because the initial state of every delay cell is zero prior to the excitation of the input sequence. We take the example of the direct-type FIR system [24]. The value of the output sample at any time depends on all or part of the input samples and the system state at that time. The first *N_f_* − 1 output samples suffer from biases, because a part of the delay cells do not yet become input-driven states; analogously, the last *N_f_* − 1 output samples are invalid, because a part of the delay cells restore the initial zero-states. Thus, the length of the valid part of the output sequence, defined as *L_υ_*, satisfies:

(4) $$L_{\upsilon }=L-2\left(N_{f}-1\right)=N-N_{f}+1=N-N_{o}$$

Actually, if we rewrite Equation (1) in the following matrix form:

(5) $$\underset{\text{y}}{\underset{︸}{\left[\begin{array}{c} y_{0}\\ y_{1}\\ \vdots \\ y_{L-1} \end{array}\right]}}=\underset{\text{X}}{\underset{︸}{\left[\begin{array}{cccc} x_{0} & 0 & \cdots & 0\\ x_{1} & x_{0} & \ddots & 0\\ \vdots & \vdots & & \vdots \\ x_{N-1} & x_{N-2} & \ddots & x_{0}\\ 0 & x_{N-1} & & x_{1}\\ \vdots & \vdots & \ddots & \vdots \\ 0 & 0 & \cdots & x_{N-1} \end{array}\right]}}\underset{\text{h}}{\underset{︸}{\left[\begin{array}{c} h_{0}\\ h_{1}\\ \vdots \\ h_{N_{o}} \end{array}\right]}}$$

then we can find that the matrix **X** possesses many zero elements, which probably makes the outputs *y*_0_, *y*_1_, …, *y*_*N_o_*−1_; *y*_*L*−*N_o_*_, *y*_*L*−*N_o_*__+1_, _…_, *y*_*L*−1_ not ideal. For example, *y*_0_ = *x*_0_*h*_0_, while the ideal output should be *ỹ*_0_ = *x*_0_*h*_0_ + *x*_−1_*h*_1_ + *x*_−2_*h*_2_ + ⋯ + *x*_−*N_o_*_*h*_*N_o_*_. This means that the unknown samples *x*_−*N_o_*_, *x*_−*N_o_*+1_, _…_, *x*_−1_ are assumed to be zeros. The filtering error of *y*_0_ is *y*_0_ − *ỹ*_0_ = −(*x*_−1_*h*_1_ + *x*_−2_*h*_2_ + ⋯ + *x*_−*N_o_*_*h_N_o__*). Likewise, the outputs *y*_1_, *y*_2_, …, *y*_*N_o_*−1_; *y*_*L*−*N_o_*_, *y*_*L*−*N_o_*__+1_, _…_, *y*_*L*−1_ all suffer from errors under the zero assumption. Thus, we can conclude that the meaningless zeros are the error sources of the filtering.

Referring to Equation (4), we note that, if the filter order is not less than the length of the input sequence, the output samples are all invalid. Thus, improving filtering performance by means of unlimited increasing filter order is meaningless.

In the next subsection, we will identify an efficient way to resolve this problem.

### 2.2. Filtering Procedure Based on Signal Extrapolation

The desired output of the filtering process should have two characteristics:

- The original and filtered sequences should be of equal length;
- During the filtering process, the states of the delay cells in the filter system should always maintain input-driven states, *i.e.*, there are no artificial zeros, but authentic samples in **X**.

**Figure 1.**
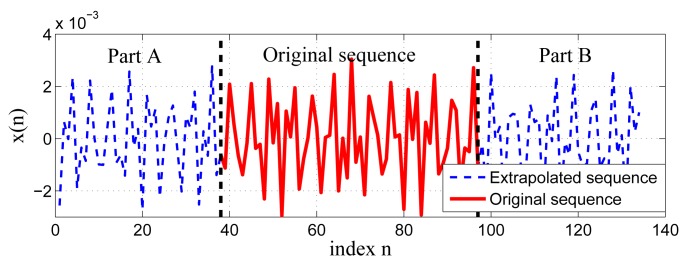
Original sequence and its extrapolated sequence.

As shown in Equation (5), the convolution filtering assumes the unknown samples *x*_−*N_o_*_, *x*_−*N_o_*+1_, _…_, *x*_−1_; *x_N_*, *x*_*N*+1_, _…_, *x*_*N*+*N_o_*−1_ to be zeros, which leads to the filtering error. Thus, an intuitive thought is to extrapolate the sequence ${\left\{x_{n}\right\}}_{\left(n=0\right)}^{\left(N-1\right)}$ along two sides to provide a series of estimates for the unknown samples. Taking place of the zeros in the matrix **X** with these estimates can mitigate the filtering error. The input sequence is extrapolated along both sides, yielding two extrapolated sequences, called Part A and Part B (see Figure 1). Suppose that *L_A_* and *L_B_* are the lengths of Part A and Part B, respectively; then, those *L_A_* + *L_B_* extrapolated samples are used to replace zeros in **X**. According to Equation (3), the length of the associated output sequence is *L_A_* + *L_B_* + *N* + *N_o_*. From Equation (4), the effective length of the output can be given by *L_A_* + *L_B_* + *N* − *N_o_*. To satisfy the requirement that the original and filtered sequence are of equal length, the extrapolated length can be derived as:

(6) $$L_{A}+L_{B}+N-N_{o}=N\Rightarrow L_{A}+L_{B}=N_{o}$$

Now, we can conclude that the extrapolated length should be equal to the filter order. We define *L_G_* as the constant group delay of the filter. Between time *N_o_* and time *N* + *N_o_*, the output samples are valid. The output sample at time *N_o_* + *n* (*n* = 1, 2, ⋯ , *N*) corresponds to the input sample at time *N_o_* − *L_G_* + *n* (*n* = 1, 2, ⋯ , *N*), because of the group delay. Consequently, the input sample before time *N_o_* − *L_G_* is merely used as a training sequence of the system state. Thus, we can obtain the relationships:

(7) $$\{\begin{array}{l} L_{A}=N_{o}-L_{G}\\ L_{B}=L_{G} \end{array}$$

Let *x̂_n_* and *ŷ_n_* be the extrapolated sequence and associated filtered result, respectively. Then, they satisfy:

(8) $$\{\begin{array}{cc} \hat{x_{n}}: & L_{G}-N_{o}⩽n⩽N+L_{G}-1\\ h_{n}: & 0⩽n⩽N_{o}\\ \hat{y_{n}}: & L_{G}⩽n⩽L_{G}+N-1 \end{array}$$

(9) $${\hat{x}}_{n}=x_{n}\left(0⩽n⩽N-1\right)$$

The filtering process can be rewritten in matrix form as:

(10) $$ŷ=\hat{\text{X}}\text{h}$$

where:

(11) $$ŷ={\left[{ŷ}_{L_{G}},{ŷ}_{L_{G}+1},\ldots ,{ŷ}_{L_{G}+N-1}\right]}_{\left(N\times 1\right)}^{\text{T}}$$

and:

(12) $$\hat{\text{X}}={\left[\begin{array}{cccc} {\hat{x}}_{L_{G}} & {\hat{x}}_{L_{G}-1} & \ldots & {\hat{x}}_{L_{G}-N_{o}}\\ {\hat{x}}_{L_{G}+1} & {\hat{x}}_{L_{G}} & \ldots & {\hat{x}}_{L_{G}-N_{o}+1}\\ \vdots & \vdots & \ddots & \vdots \\ {\hat{x}}_{L_{G}+N-1} & {\hat{x}}_{L_{G}+N-2} & \ldots & {\hat{x}}_{L_{G}+N-N_{o}-1} \end{array}\right]}_{\left(N\times N_{f}\right)}$$

### 2.3. Signal Extrapolation Based on AR Identification and Kalman Prediction

According to the linear prediction theory [25], the AR model is an all-pole model, whose output variable only linearly depends on its own previous values, that is,

(13) $$\{\begin{array}{l} \Phi \left(q^{-1}\right)x_{n}={ɛ}_{n}\\ \Phi \left(q^{-1}\right)=\overset{p}{\underset{l=0}{\text{∑}}}\phi_{l}q^{-l} \end{array},n=0,1,\cdots ,N-1$$

where *q*^−1^ denotes the unit delay, *p* is the model order, *ϕ*_0_, *ϕ*_1_, …, *ϕ_p_* denote the coefficients of the model and *ϕ*_0_ = 1. The sequence ${\left\{{ɛ}_{n}\right\}}_{n=-\infty }^{\infty }$ is a white noise process, which satisfies:

(14) $$\{\begin{array}{ll} E\left({ɛ}_{n}\right)=0, & \forall n\\ E\left({ɛ}_{n}^{2}\right)=\sigma^{2}, & \forall n\\ E\left({ɛ}_{n}{ɛ}_{n^{\prime }}\right)=0, & n\neq n^{\prime } \end{array}$$

where *E* (·) denotes the expectation operator.

We choose the forward-backward approach [1] as the coefficient estimator for AR model, for its precision and robustness. Both criteria, including the Akaike information criterion (AIC) and Bayesian information criterion (BIC) [26] can be applied to determine the model order; whereas both criteria sometimes suffer from overfitting. An alternative method of order determination will be discussed in Section 2.4.

A linearly-optimal prediction for AR sequences is derived in [21–23] under the minimum mean square error (MMSE) criterion. However, the prediction formula involves a polynomial long division and a coefficient polynomial recursion [23], making the calculation of the prediction somewhat inconvenient. Alternatively, the following steady-state Kalman predictor [27] provides an equivalent prediction with the MMSE predictor, while offering a simpler formula to facilitate the computation.

The AR model is regarded as a dynamic system. A specific state-space representation for a univariate AR(p) process can be written as [25]:

(15) $$\{\begin{array}{c} \xi_{n+1}=\text{F}\xi_{n}+\Gamma {ɛ}_{n}\\ x_{n}=\text{H}\xi_{n}+{ɛ}_{n} \end{array}$$

where:

(16) $$\text{F}={\left[\begin{array}{ccccc} -\phi_{1} & 1 & 0 & \cdots & 0\\ -\phi_{2} & 0 & 1 & \cdots & 0\\ \vdots & \vdots & \vdots & \cdots & 0\\ -\phi_{p-1} & 0 & 0 & \cdots & 1\\ -\phi_{p} & 0 & 0 & \cdots & 0 \end{array}\right]}_{\left(p\times p\right)}$$

(17) $$\Gamma ={\left[-\phi_{1}-\phi_{2}\cdots -\phi_{p-1}-\phi_{p}\right]}_{\left(p\times 1\right)}^{\text{T}}$$

and:

(18) $$\text{H}={\left[1\;0\;\cdots \;0\right]}_{\left(1\times p\right)}$$

The coefficient polynomials of *x_n_* and *ε_n_* are Φ(*q^−^*^1^) and one, respectively. Since they are relative prime polynomials (or coprime), *i.e.*, the transfer function is irreducible, the system of the AR model is a joint controllable and observable discrete linear stochastic system [28]. Thus, there exists a steady-state Kalman predictor:

(19) $$\{\begin{array}{c} {\hat{\xi }}_{n+1|n}=F{\hat{\xi }}_{n|n-1}+\text{K}e_{n}\\ x_{n}=\text{H}{\hat{\xi }}_{n|n-1}+e_{n} \end{array}$$

Since both *ε_n_* and *e_n_* are the innovation processes of *x_n_*, they are equal [27]:

(20) $$e_{n}={ɛ}_{n}$$

By comparing Equation (15) with Equation (19), we have:

(21) $$\{\begin{array}{ll} \xi_{n} & =\xi_{n|n-1}\\ \text{K} & =\Gamma \end{array}$$

Therefore, the one-step steady-state Kalman predictor can be derived as [28]:

(22) $$\begin{array}{ll} {\hat{x}}_{n+1|n} & =H{\hat{\xi }}_{n+1|n}=x_{n+1}-{ɛ}_{n+1}\\ & =x_{n+1}-\Phi \left(q^{-1}\right)x_{n+1}=-\overset{p}{\underset{l=1}{\text{∑}}}\phi_{l}x_{n-l+1} \end{array}$$

Similarly, the *k*-step steady-state Kalman predictor can be presented as:

(23) $$\{\begin{array}{l} {\hat{\xi }}_{n+k|n}=F{\hat{\xi }}_{n+k-1|n-1}+F^{k-1}\Gamma {ɛ}_{n}\\ {\hat{x}}_{n+k|n}=\text{H}{\hat{\xi }}_{n+k|n} \end{array}$$

Here, define:

(24) $$\{\begin{array}{c} {\text{F}}^{k-1}\Gamma ={\left[g_{k_{0}},g_{k1},\cdots ,g_{k_{p-1}}\right]}^{\text{T}}\\ G_{k}\left(q^{-1}\right)=g_{k_{0}}+g_{k_{1}}q^{-1}+\cdots +g_{k_{p-1}}q^{-\left(p-1\right)} \end{array}$$

Then, we can obtain:

(25) $$\Phi \left(q^{-1}\right){\hat{x}}_{n+k|n}=G_{k}\left(q^{-1}\right){ɛ}_{n}$$

and that is,

(26) $${\hat{x}}_{n+k|n}=G_{k}\left(q^{-1}\right)x_{n}=\overset{p-1}{\underset{l=0}{\text{∑}}}g_{k_{l}}x_{n-l}$$

Analogously, the *k*-step backward extrapolation formula can be given by:

(27) $${\hat{x}}_{n-k|n}=\sum_{l=0}^{p-1}g_{k_{l}}^{*}x_{n+l}$$

where the superscript “*” denotes the complex conjugate operator. To guarantee reasonable and effective extrapolations, the step-size *k* should satisfy:

(28) $$\{\begin{array}{l} L_{G}-N_{o}+k⩾0\\ N+L_{G}-1-k⩽N-1 \end{array}\Rightarrow L_{G}⩽k⩽N_{o}-L_{G}$$

In order to evaluate the residual filtering error of the proposed filtering strategy, we derive the mean square error (MSE) in Appendix A1.

### 2.4. Adaptive Information Criteria for AR Order Determination

Given the impulse response of an analysis filter and AR coefficients, we can directly calculate MSE by Equations (A2) and (A10). The precision of AR coefficient estimation is concerned with AR order. Consequently, the filtering error at different AR orders can be evaluated with the preceding formulas; conversely, the calculation of MSE can be used for order determination.

AIC and BIC are two common information criteria, whose purpose is to find a model with sufficient goodness of fit and a minimum number of free parameters. In terms of the maximum likelihood estimate ${\hat{\sigma }}_{p}^{2}$ we can denote AIC and BIC as [26]:

(29) $$\underset{P}{\min}\text{AIC}=\log\left({\hat{\sigma }}_{p}^{2}\right)+\frac{2\left(p+1\right)}{N}$$

(30) $$\underset{P}{\min}\text{BIC}=\log\left({\hat{\sigma }}_{p}^{2}\right)+\frac{p\log N}{N}$$

**Figure 2.**
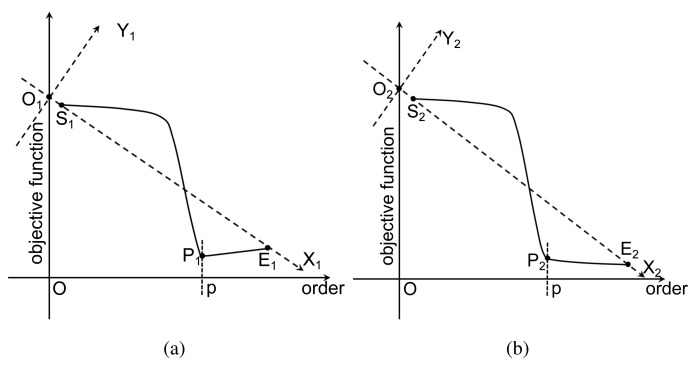
Geometric interpretation for adaptive Akaike information criterion (AAIC) and adaptive Bayesian information criterion (ABIC): the solid curves $\tilde{{\text{S}}_{1}{\text{P}}_{1}{\text{E}}_{1}}$ and $\tilde{{\text{S}}_{2}{\text{P}}_{2}{\text{E}}_{2}}$ draw objective function values for AIC or BIC. The preferred orders are located at the point P_1_ and P_2_, respectively. (**a**) The case that the criterion (AIC or BIC) successfully determines the correct order, and (**b**) the case that the criterion fails due to the inadequate penalty strength. Under the new coordination system X_2_O_2_Y_2_, the point P_2_ becomes the minimum point of the curve, and the correct order is retrieved.

As explained in [29], due to the lack of samples, both criteria encounter the risk of overfitting, where the selected order will be larger than the truth order. In particular, AIC has the nonzero overfitting probability as the sample number tends to infinity. Theoretically, both criteria consist of two terms: the first term involves MSE, and it decreases with the increment of the order *p;* the other term is a penalty that is an increasing function *of p.* The preferred model order is the one with the lowest AIC or BIC value. As shown in Figure 2a, the objective function curve $\tilde{{\text{S}}_{1}{\text{P}}_{1}{\text{E}}_{1}}$ reaches its minimum value at the point P_1_, which gives the correct order *p.* However, sometimes, both criteria may fail to determine available orders, and those failures are often related to inadequate penalties. Figure 2b illustrates a representative case. Since the change of the objective function instantly slows down as the order exceeds *p*, the point P_2_ is the preferred point for order determination. However, the penalty strength is insufficient, so that the objective function is still falling after P_2_. To handle this situation, we propose an adaptive mechanism to adaptively adjust the penalty strength. A geometric interpretation is depicted in Figure 2b. We assume that the order interval for computation consists of the correct order. Then, the ray $\vec{{\text{S}}_{2}{\text{E}}_{2}}$ forms the X_2_ axis, while the ray $\vec{{\text{O}}_{2}{\text{Y}}_{2}}$ forms the Y_2_ axis perpendicular to the ray $\vec{{\text{S}}_{2}{\text{E}}_{2}}$ throughout the intersection O_2_ of the ray $\vec{{\text{S}}_{2}{\text{E}}_{2}}$ and the objective function axis. Under the new coordination system X_2_O_2_Y_2_, the minimum point P_2_ of the curve $\tilde{{\text{S}}_{2}{\text{P}}_{2}{\text{E}}_{2}}$ can help to determine the correct order. Meanwhile, this modification has no impact on the case that the criterion works well (see Figure 2a).

Therefore the adaptive AIC (AAIC) based on MSE of the residual filtering error can be derived as:

(31) $$\{\begin{array}{l} \underset{p}{\min}\text{AAIC}=\log\left({\hat{\sigma }}_{p}^{2}\right)+\frac{2}{N}\left(p^{\alpha }+1\right)\\ s.t.\text{AAIC}\left(p_{s}\right)=\text{AAIC}\left(p_{e}\right) \end{array}$$

where *p_s_* and *p_e_* denote the start point and the end point of the computing order interval, respectively. If *p_s_*= 1 the adaptive parameter *α* can be given by

(32) $$\alpha =\log_{p_{e}}\left[\frac{N}{2}\log\left(\frac{{\hat{\sigma }}_{1}^{2}}{{\hat{\sigma }}_{p_{e}}^{2}}\right)+1\right]$$

Analogously, the adaptive BIC (ABIC) can be represented as:

(33) $$\{\begin{array}{l} \underset{p}{\min}\text{ABIC}=\log\left({\hat{\sigma }}_{p}^{2}\right)+p^{\beta \frac{\log N}{N}}\\ \beta =\log_{p_{e}}\left[\frac{N}{\log N}\log\left(\frac{{\hat{\sigma }}_{1}^{2}}{{\hat{\sigma }}_{p_{e}}^{2}}\right)+1\right] \end{array}$$

## 3. Implementation of SDSE

In this section, we discuss the implementation details of SDSE based on the proposed filtering strategy. In particular, equiripple FIR filters are used as the analysis filters for their advantageous features. To suppress spectral overlap and improve spectral precision in practice, we introduce a mosaicking operation for sub-band spectra and discuss the compensation of the residual error of the composite spectrum. After that, we summarize the entire algorithm and analyze the computational complexity.

### 3.1. Properties and Design of Equiripple FIR Filters

**Figure 3.**
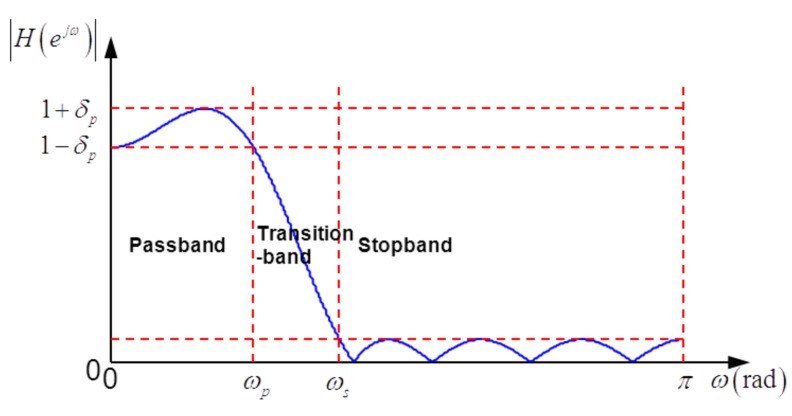
Magnitude response and design parameters of an equiripple low-pass FIR filter.

Besides the advantages of FIR filters, *i.e.*, exact linear phase response and inherent stabilization, equiripple FIR filters have an explicitly specified transition width and passband/stop-band ripples (see Figure 3). As analysis filters, equiripple FIR filters can bring some important benefits, such as stop-band attenuation with a fixed maximum, the explicitly specified width of the invalid part of the sub-band spectrum (which corresponds to the transition-band spectrum) and a limited maximum deviation of the valid part of the sub-band spectrum (which corresponds to the passband spectrum). As shown in Figure 3, the specifications of a typical equiripple FIR filter consist of the passband edge *ω_p_*, stop-band edge *ω_s_* and maximum error in passband and stop-band *δ_p_, δ_s_*, respectively. The approximate relationship between the optimal filter length and other parameters developed by Kaiser [11] is:

(34) $$N_{f}\approx \frac{-20\log_{10}\left(\sqrt{\delta_{p}\delta_{s}}\right)-13}{14.6\Delta f}+1$$

where Δ*f* denotes the width of the transition-band,

(35) $$\Delta f=\frac{\omega_{s}-\omega_{p}}{2\pi }$$

The maximum passband variation and the minimum stop-band attenuation in decibels are defined as:

(36) $$A_{p}=20\log_{10}\left(\frac{1+\delta_{p}}{1-\delta_{p}}\right)\text{dB}$$

and:

(37) $$A_{s}=-20\log_{10}\left(\delta_{s}\right)\text{dB}$$

respectively.

When the specification of a filter is explicitly specified, we can complete the design with the Parks–McClellan (PM) algorithm [30], since it is optimal with respect to the Chebyshev norm and results in about 5 dB more attenuation than the windowed design algorithm [11].

### 3.2. Practical Consideration of Equiripple FIR Filters

**Figure 4.**
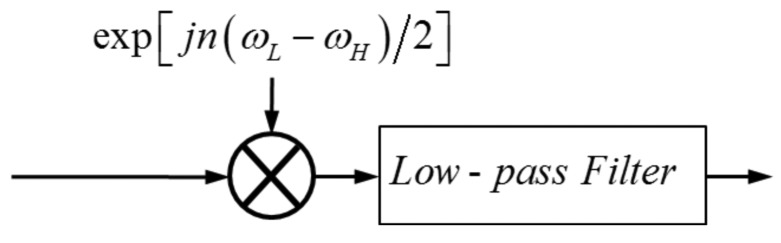
Block diagram of the analysis filter.

Firstly, the equiripple low-pass FIR filter is combined with a preprocessing step—complex frequency modulation—to form a passband filter for sub-band decomposition (see Figure 4).

**Figure 5.**
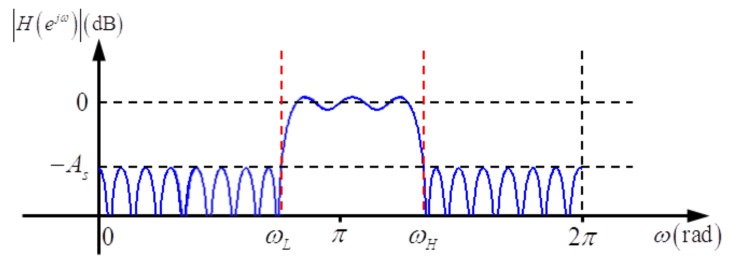
Magnitude response of the analysis filter.

The magnitude response of the analysis filter is shown in Figure 5, where *ω_H_* and *ω_L_* denote the high and the low edge of the stop-band, respectively. They satisfy:

(38) $$\omega_{H}-\omega_{L}=2\omega_{s}$$

As long as *A_s_* is large enough and the downsample rate *M* meets the condition:

(39) $$M⩾\frac{2\pi }{\omega_{H}-\omega_{L}}\Rightarrow M⩾\frac{\pi }{\omega_{s}}$$

frequency aliasing can be practically suppressed.

**Figure 6.**
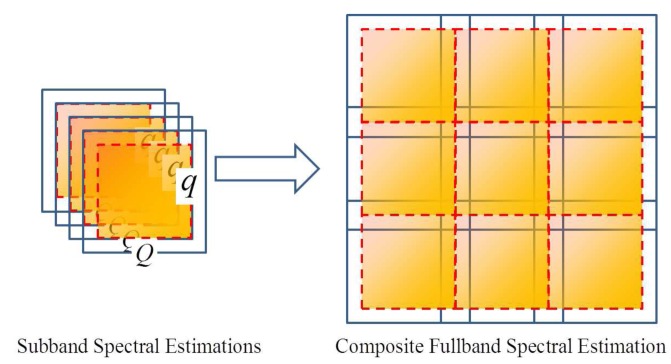
Illustration of mosaicking the sub-band spectral estimations into a composite spectrum. The sub-band spectral estimations are overlapped, while the composite full-band spectrum is without overlap (the boxes with solid lines cover the spectral estimation of the sub-bands; the boxes with dashed lines cover the valid spectral estimation).

Secondly, due to the existence of the transition-band of each analysis filter, each sub-band spectrum contains two invalid parts. The spectral estimations of these invalid parts lead to errors. Consequently, according to [31], when mosaicking these sub-band spectral estimations into full-band, we should omit these invalid parts of spectral estimations. This procedure is illustrated in Figure 6. Thus, the composite full-band spectral estimation is practically immune to the spectral overlap.

Thirdly, due to the existence of passband ripples in equiripple FIR filters, there theoretically remains a small error in sub-band spectral estimations. Generally, by adjusting the maximum passband variation, we can limit the error to an allowable range. More precise spectral estimation necessitates compensation for the residual error. Since the ripple curve for any given equiripple FIR filter can be accurately measured, the compensation can be performed by weighting sub-band spectral estimations with the measured ripple curve.

Finally, we focus on selecting appropriate filter parameters in SDSE, which can improve the performance and reduce computational cost. The filter order should at least meet:

(40) $$N⩾\text{max}\left(L_{G},N_{o}-L_{G}\right)$$

The maximum stop-band attenuation should exceed the dynamic range of the signal to be analyzed. Once the aforementioned conditions are satisfied, the shortest transition-width can be chosen by Equation (34). Moreover, specific requirement will help to set the maximum passband variation.

### 3.3. Computational Complexity of SDSE


**Algorithm 1** Non-overlapping sub-band spectral estimation with the steady-state Kalman predictor-based filtering strategy.
**Input:** The sequence 
${\left\{x_{n}\right\}}_{n=0}^{N-1}$.**Parameters:** The maximum passband variation *A_p_* and the minimum stop-band attenuation *A_s_* in decibels; the sub-band number *M*.**Filter Design:** Set the stop-band edge *ω_s_* by Equation (39); Set the passband edge *ω_p_* by Equations (34), (35), (36), (37) and (40); Design the equiripple FIR filter by Parks–McClellan (PM) algorithm [30], and then, compute the impulse response 
${\left\{h_{n}\right\}}_{n=0}^{N_{o}}$ and the group delay *L_G_*.**AR Identification and Order Selection:** **for**
*p_i_* = *p_s_* to *p_e_* (usually set *p_s_* = 1, *p_e_* ⩽ *N*/2 − 1) **do**  Estimate coefficients 
${\left\{\phi_{i}\right\}}_{i=1}^{p_{i}}$ of AR model by the forward and backward estimator, with 

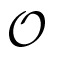

$\left(Np_{i}^{2}\right)$ flops;  Estimate the MSE 
${\hat{\sigma }}_{p_{i}}^{2}$ by (A10) and (A11), with 

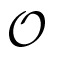

$\left(\frac{N_{o}^{4}}{48}+\frac{5}{24}N_{o}^{3}+\frac{N_{o}^{2}}{6}\right)$ flops; **end for** Select an order *p* by Equation (31) or Equation (33), with 

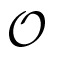
 (*N*) flops.**Sequence Extrapolation:** Set the step-size *k* by Equation (28); Calculate 
${\left\{g_{k_{l}}\right\}}_{l=0}^{p-1}$ by Equations (16), (17) and (24), with 

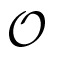
 ((2*p*)*^k^*^−1^) flops; Implement forward and backward extrapolations by Equations (26) and (27), and obtain 
${\left\{{\hat{x}}_{n}\right\}}_{n=L_{G}-N_{o}}^{L_{G}+N-1}$ , with 

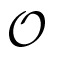
 (*N_o_ p*) flops.**Sub-Band Spectral Estimation:** Set a rational factor 
$M_{0}=〚\frac{\pi }{\omega_{s}}〛$, where [[]] denotes a rational approximation; **for**
*i* = 1 to *M*
**do**  Compute *ω_H_* and *ω_L_* by Equation (38) and *ω_H_* + *ω_L_* = (2*i* − 1) π/*M*;  Perform pre-modulation and filtering for 
${\left\{{\hat{x}}_{n}\right\}}_{n=L_{G}-N_{o}}^{L_{G}+N-1}$ by Figure 4 and Equation (10), and the computational complexity is in 

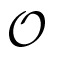
 (2 (*N* + *N_o_*) log (*N + N_o_*)) flops;  Decimate the sequence 
${\left\{{\hat{x}}_{n}\right\}}_{n=L_{G}-N_{o}}^{L_{G}+N-1}$, by a factor of *M*_0_, and obtain the sub-band sequence 
${\left\{{\hat{x}}_{n}^{\left(i\right)}\right\}}_{n=0}^{\left\lceil \frac{N}{M_{0}}\right\rceil -1}$ , where ⌈ ⌉ denotes the ceiling function;  Perform spectral analysis for the sub-band sequence 
${\left\{{\hat{x}}_{n}^{\left(i\right)}\right\}}_{n=0}^{\left\lceil \frac{N}{M_{0}}\right\rceil -1}$, and denote the length of the sub-band spectrum as *L_s_*;  Compute the length of overlapped spectrum by 
$\left\lceil \left(\frac{1}{M_{0}}-\frac{\omega_{p}}{\pi }\right)\frac{L_{s}}{2}\right\rceil$ and omit the overlapped parts at both the left and the right side of the sub-band spectrum. **end for** Mosaic the residual sub-band spectrums into an entire spectrum.**Output:** The entire spectrum.


As shown in Algorithm 1, we summarize SDSE with the proposed filtering strategy and give the computational complexity of the major steps. First, the proposed strategy can greatly reduce the computational burden. We take the commonly-used amplitude and phase estimation (APES) [32] algorithm as an example. The full-band APES needs 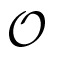 (*N*^2^ log *N*) flops [33], while the computation requirement is decreased to 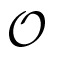 $\left(M{\left(\left\lceil \frac{N}{M_{0}}\right\rceil \right)}^{2}\log\left(\left\lceil \frac{N}{M_{0}}\right\rceil \right)\right)$ flops by SDSE with the proposed strategy. Second, except the sub-band spectral estimation, the main computation requirement is induced by the AR identification and the order selection. The computational complexity of this step is generally much lower than that of the sub-band spectral estimation. In particular, if a proper order or a small enough order interval is preselected before the AR identification, the computation of this step can be negligible.

## 4. Simulations and Analysis

In this section, both the feasibility and the effectiveness of the proposed strategy are evaluated by typical numerical simulations, including FIR filtering and line spectral analysis of 1D or 2D sequences.

### 4.1. Filtering Analysis Using the Proposed Strategy

Suppose that the input sequence {*x_n_*} is a mixed complex exponential sequence:

(41) $$\{\begin{array}{ll} x_{n} & =s_{n}^{\left(1\right)}+s_{n}^{\left(2\right)}+\upsilon_{n}\\ s_{n}^{\left(1\right)} & =\exp\left(0.45j\pi n\right)\\ s_{n}^{\left(2\right)} & =\overset{25}{\underset{l=0}{\text{∑}}}100\exp\left\{\left(0.55+0.035l\right)j\pi n\right\}\\ n & =0,1,\ldots ,127 \end{array}$$

where the measurement noise {*υ_n_*} is a complex Gaussian process. All real parts and imaginary parts of {*υ_n_*} are independent and identically distributed (i.i.d.) zero-mean Gaussian distributions with variance *σ^2^, i.e.*, Re (*υ_n_*), Im (*υ_n_*) ∼ 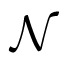 (0, *σ*^2^). Our purpose is to non-distortedly extract the weak component $s_{n}^{\left(1\right)}$ from *x_n_* or completely eliminate the strong component $s_{n}^{\left(2\right)}$.

The equiripple half-band low-pass FIR filter is chosen for the extraction. The specifications of the filter are:

(42) $$A_{p}=1.4295\times 10^{-3}\text{dB},A_{\text{s}}=81.6852\text{dB},\Delta f=0.08$$

The length of the designed filter based on the given specifications is 119.

**Figure 7.**
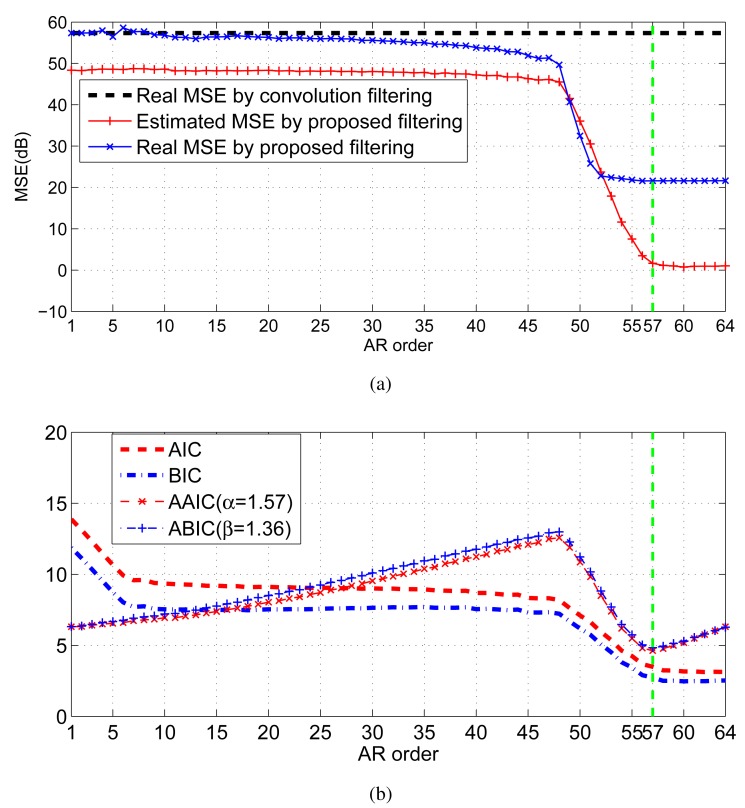
Mean square error of filtering and order selection: (**a**) quantitative comparison of the filtering error by convolution filtering and the proposed filtering; (**b**) comparison of the information criteria, including AIC, BIC, AAIC and ABIC.

As shown in Figure 7a, the decreasing trend of the estimated residual error by the proposed strategy is consistent with the real error. When the order exceeds 57, the decrease of the estimated filtering error instantly slows down. Hence, the preferred order is 57. By comparison, due to the deficiency of the penalty strength, none of AIC and BIC can provide the right order; whereas, based on the adaptive penalty terms, both AAIC and ABIC get the right order 57 (see Figure 7b).

**Figure 8.**
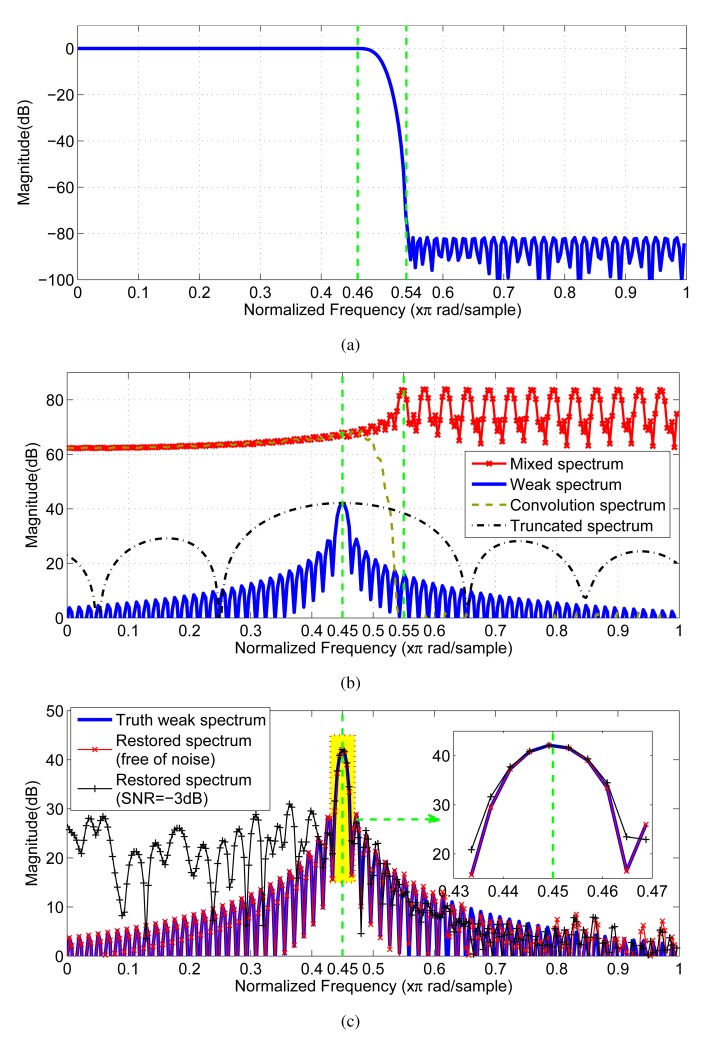
Quantitative comparison of filtering results: (**a**) magnitude response of the designed equiripple finite impulse response (FIR) half-band filter; (**b**) Fourier spectra of the mixed sequence, the truth weak component, the convolved sequence and the truncated sequence with 10 samples; (**c**) Fourier spectra of the truth weak component; the restored results by the proposed filtering strategy when the mixed sequence is free of noise or contaminated by noise (SNR = −3 dB).

As shown in Figure 8b, the weak component $s_{n}^{\left(1\right)}$ is completely covered by the sidelobe of the out-of-band strong component $s_{n}^{\left(2\right)}$ thus, recognizing the existence of the weak component from the mixed spectrum is completely impossible. From the view of the magnitude response (see Figure 8a), the filter has the nominal ability of eliminating the interference of the out-of-band strong components for the in-band weak component. Due to the existence of the convolution filtering error, we still cannot find out the weak component from the convolution spectrum, as shown in Figure 8b. By contrast, once the samples contaminated by the filtering error are omitted by Equation (4) from the filtered sequence, the weak component reappears in the spectrum of the remaining samples (refer to the truncated spectrum in Figure 8b). However, the truncated spectrum has a much wider main lobe than the original spectrum, which means the spectral resolution suffers from a severe decrease. In order to simultaneously maintain the resolution and filter out the interference, we apply the proposed filtering strategy to handle the case. As shown in Figure 8c, based on the proposed strategy, the restored spectrum for the noiseless sequence closely coincides with the truth weak spectrum in shape, especially retaining the spectral resolution. In addition, even when the signal-to-noise (SNR) of $s_{n}^{\left(1\right)}$ is low to −3 dB (when σ^2^ = 1), the recovery is still effective (see the magnified details of Figure 8c).

### 4.2. Line Spectral Analysis Using 1-D Signals

A complex exponential model can be mathematically represented as:

(43) $$\{\begin{array}{ll} x_{n} & =s_{n}^{\left(1\right)}+s_{n}^{\left(2\right)}+\upsilon_{n}\\ s_{n}^{\left(1\right)} & =\overset{5}{\underset{k=1}{\text{∑}}}\alpha_{k}\exp\left[j\left(\omega_{k}n+\phi_{k}\right)\right]\\ s_{n}^{\left(2\right)} & =\overset{16}{\underset{i=0}{\text{∑}}}100\left\{\exp\left[j\left(\omega_{i}^{\left(+\right)}n+\phi_{i}^{\left(+\right)}\right)\right]+\exp\left[j\left(\omega_{i}^{\left(-\right)}n+\phi_{i}^{\left(-\right)}\right)\right]\right\} \end{array}$$

(43) where:

$$\begin{array}{c} \alpha_{1}=\alpha_{3}=\alpha_{5}=5,\alpha_{2}=\alpha_{4}=1\\ \omega_{1}=-0.075\pi ,\omega_{2}=-0.03125\pi ,\omega_{3}=0.0125\pi \omega_{4}=0.05625\pi \\ \omega_{5}=0.1\pi ,\omega_{i}^{\left(+\right)}=\left(0.15+0.05i\right)\pi ,\omega_{i}^{\left(-\right)}=-\left(0.15+0.05i\right)\pi \end{array}$$

and:

n=0,1,⋯,N−1;N=128

{*υ_n_*} is a real-value sequence of i.i.d. zero-mean Gaussian random variables with variance *σ*^2^ = 1.5811, *i.e., u_n_* ∼ 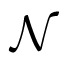 (0, σ^2^). *ϕ_k_*, $\phi_{i}^{+}$ and $\phi_{i}^{-}$ are i.i.d. uniform random variables on the interval from zero to 2*π, i.e.*, *ϕ_k_*, $\phi_{i}^{\left(+\right)}$, $\phi_{i}^{\left(-\right)}$ ∼ 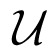 [0, 2*π*).

In this case, we can get each component's SNR of $s_{n}^{\left(1\right)}$:

$${\text{SNR}}_{1}={\text{SNR}}_{3}={\text{SNR}}_{5}{=5\text{dB},\text{SNR}}_{2}={\text{SNR}}_{4}=-2\text{dB}$$

We decompose the mixed-signal *x_n_* into four sub-bands using the proposed method with the filter parameter set as:

(44) $$A_{p}=0.01\text{dB},A_{s}=60\text{dB},\Delta f=0.05$$

The sub-band, whose radian frequency is within [−0.125*π*, +0.125*π*), is used for frequency estimation. Furthermore, we estimate the frequencies of complex sinusoids of $s_{n}^{\left(1\right)}$ that are contained in both mixed-signal *x_n_* and the decomposed sub-band signal, via MUSIC, ESPRIT [34,35] and SELF-SVD [15] algorithms (see Table 1). As shown in Table 1, we analyze the performance based on the Monte Carlo method. Compared with ESPRIT, SELF-SVD in full-band spectral estimation suffers from obvious performance degradations or even failures. Although SELF-SVD can theoretically attenuate the out-of-band components for the in-band frequency estimations, the ability of attenuation is not always sufficient, especially when the power of the out-of-band components are much stronger than that of the in-band components or the SNR is relatively low. Instead of performing the SVD method in the entire frequency domain as ESPRIT, SELF-SVD just performs it in the frequency interval of interest. Obviously, the remaining out-of-band interferences will be treated as in-band components, so that the frequency estimation with SELF-SVD sometimes fails. In the experiment, the power ratio of the out-of-band components to the in-band components *ω*_2_ and *ω*_4_ is up to 10,000 times. As a result, the corresponding frequency estimation with SELF-SVD fails to work. When we eliminate the out-of-band interferences with our method, the estimation of SELF-SVD for the residual signal exhibits similar performance as ESPRIT. In addition, MSEs of MUSIC and ESPRIT indicate that the frequency estimation in the sub-band is much more accurate than that in the full-band.

### 4.3. Line Spectral Analysis Using 2D signals

Let *C_k_* (*k* = 1, 2, …, *K*) be a series of random integers with unique values generated from a uniform discrete distribution on [1,1024 × 1024]. We define two sets of nonnegative integers as:

(45) $$\{\begin{array}{l} p_{k}=\left\lfloor \frac{C_{k}}{1024}\right\rfloor \\ q_{k}=\mathrm{mod}\left(C_{k},1024\right) \end{array}k=1,2,\cdots ,K$$

where ⌊·⌋ rounds a number to the nearest integer toward zero, and mod (·) is the modulo operator.

The 2D signal model can be expressed as:

(46) $$\{\begin{array}{ll} x_{n_{1},n_{2}} & =s_{n_{1},n_{2}}^{\left(1\right)}+s_{n_{1},n_{2}}^{\left(2\right)}+\upsilon_{n_{1},n_{2}}\\ s_{n_{1},n_{2}}^{\left(1\right)} & =\overset{K}{\underset{k=1}{\text{∑}}}\exp\left[j2\pi \left(\frac{n_{1}p_{k}}{4N_{1}}+\frac{n_{2}q_{k}}{4N_{2}}\right)+j\phi_{k}\right]\\ s_{n_{1},n_{2}}^{\left(2\right)} & =\overset{16}{\underset{l_{1}=0}{\text{∑}}}1,000\left\{\begin{array}{c} \exp\left[j2\pi \left(\frac{118.5n_{1}}{N_{1}}+\frac{\left(95.5+4l_{1}\right)n_{2}}{N_{2}}\right)+j\phi_{l_{1}}^{\left(1\right)}\right]\\ +\exp\left[j2\pi \left(\frac{54.5n_{1}}{N_{1}}+\frac{\left(95.5+4l_{1}\right)n_{2}}{N_{2}}\right)+j\phi_{l_{1}}^{\left(2\right)}\right] \end{array}\right\}\\ & +\overset{15}{\underset{l_{2}=1}{\text{∑}}}1,000\left\{\begin{array}{c} \exp\left[j2\pi \left(\frac{\left(54.5+4l_{2}\right)n_{1}}{N_{1}}+\frac{95.5n_{2}}{N_{2}}\right)+j\phi_{l_{2}}^{\left(1\right)}\right]\\ +\exp\left[j2\pi \left(\frac{\left(54.5+4l_{2}\right)n_{1}}{N_{1}}+\frac{159.5n_{2}}{N_{2}}\right)+j\phi_{l_{2}}^{\left(2\right)}\right] \end{array}\right\} \end{array}$$

where:

$$n_{1}=0,1,\cdots ,N_{1}-1;n_{2}=0,1,\cdots ,N_{2}-1$$

and:

$$N_{1}=N_{2}=256,K=8,192$$

{*υ*_*n*_1_,*n*_2__} is a real-value sequence following *υ*_*n*_1_,*n*_2__ ∼ 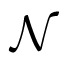 (0, σ^2^) with σ^2^ = 0.005. $\phi_{k,}\phi_{l_{1}}^{\left(1\right)},\phi_{l_{1}}^{\left(2\right)},\phi_{l_{2}}^{\left(1\right)},\phi_{l_{2}}^{\left(2\right)}$are uniform random variables on the interval from zero to 2*π, i.e.*, $\phi_{k,}\phi_{l_{1}}^{\left(1\right)},\phi_{l_{1}}^{\left(2\right)},\phi_{l_{2}}^{\left(1\right)},\phi_{l_{2}}^{\left(2\right)}∼𝒰[0,2\pi )$.

**Figure 9.**
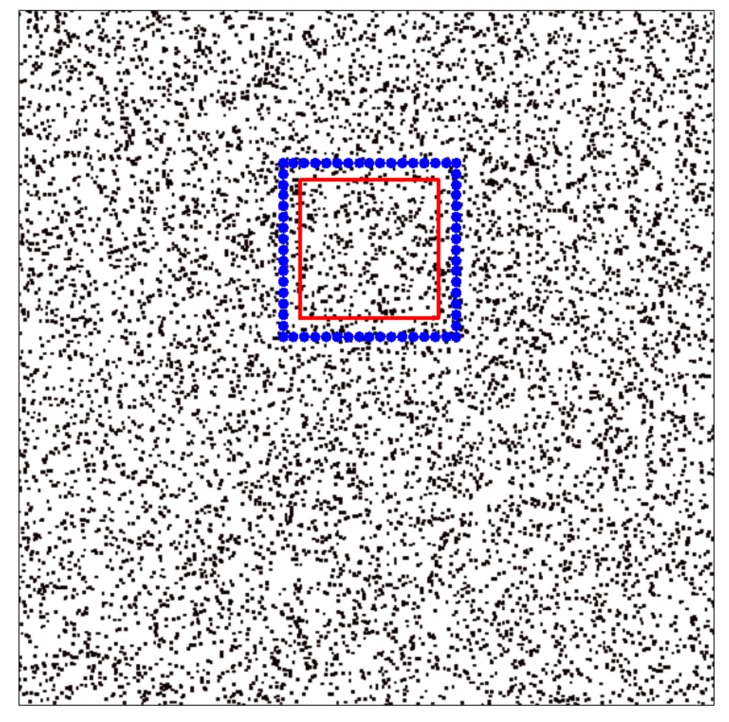
Actual magnitude spectrum of the 2D signal (the black dot corresponds to $s_{n_{1},n_{2}}^{\left(1\right)}$: 0 dB; the rounded blue spot 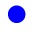 corresponds to $s_{n_{1},n_{2}}^{\left(2\right)}$: 60 dB; the red pane covers the region to be analyzed).

The spectrum of this 2D sequence is shown in Figure 9. Since the magnitude of $s_{n_{1},n_{2}}^{\left(2\right)}$ is 60 dB greater than that of $s_{n_{1},n_{2}}^{\left(1\right)}$, the sidelobe of the former significantly affects the spectral estimation of the latter. This affect is especially more severe for the components around $s_{n_{1},n_{2}}^{\left(1\right)}$. The region inside the red pane is used to verify the performance of the proposed method.

The parameters of the analysis filter are selected as:

(47) $$A_{p}=0.2\text{dB},A_{s}=80\text{dB},\Delta f=0.05$$

**Figure 10.**
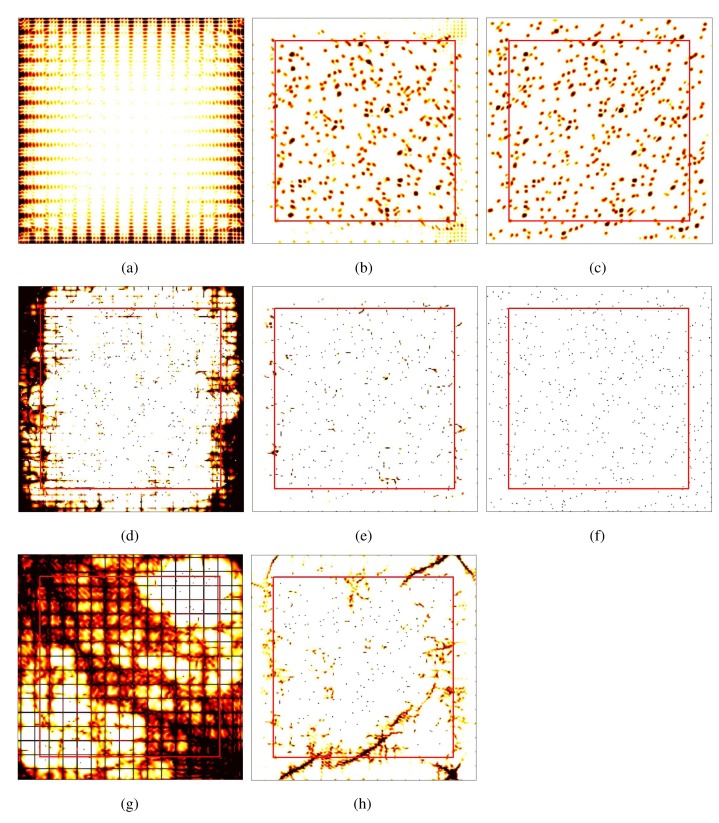
Sub-band decomposition and spectral estimation within the analyzed region: (**a**) the Fourier spectrum of *x*_*n*_1,_*n*_2__; (**b**) the Fourier spectrum of decomposed sub-band signal based on our method; (**c**) the Fourier spectrum of $s_{n_{1},n_{2}}^{\left(1\right)}$; (**d**) the amplitude and phase estimation (APES) result of (a) corresponding to the ideal frequency domain filters-based sub-band decomposition; (**e**) the APES result of (b); (**f**) the actual spectrum; (**g**) the iterative adaptive approach (IAA) result of (a); (**h**) the IAA result of (b).

The comparison of Figure 10a and 10c indicates that the Fourier spectrum of $s_{n_{1},n_{2}}^{\left(1\right)}$ is severely affected by $s_{n_{1},n_{2}}^{\left(2\right)}$. By contrast, the result shown in Figure 10b seems to be almost exactly the same as the desired result shown in Figure 10c. This decomposition result verifies the effectiveness of the proposed method.

To further testify the performance of our method, we select the APES [32] and the iterative adaptive approach (IAA) [36,37] for spectral estimation. Since the ideal frequency domain filters suffer from energy leakage and/or frequency aliasing problems, the APES result shown in Figure 10c is somewhat blurred. By contrast, the APES result of the decomposed sub-band based on the proposed strategy (see Figure 10d) is quite similar to the actual spectrum (see Figure 10e). Theoretically, the IAA is superior to the APES. However, as shown in Figure 10g, it is even more likely than the APES to suffer from out-of-band interferences. From the view of the sub-band IAA spectrum (see Figure 10h), most of the interferences are eliminated, while the remaining filtering error still has impacts on the spectrum. Thus, the spectral estimation experiment reveals that the sub-band decomposition based on the proposed method can provide relatively ideal performance; whereas the developed method for extrapolation is imperfect, so it can affect the performance of the IAA algorithm.

**Figure 11.**
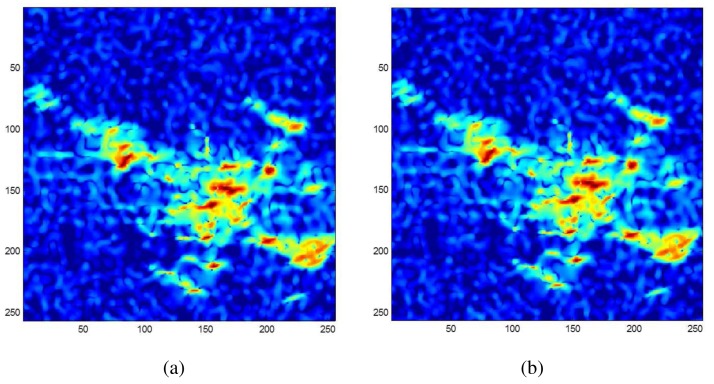
Comparison between full-band (**a**) and sub-band (**b**) APES images: the imaging for (a) costs 26.85 h, while for (b), it is 0.84 h.

In addition, a simulated single-polarized SAR image of an airplane based on the physical and optical model is processed *via* the APES. The computation time of full-band APES (refer to Figure 11a) is 26.85 h, while the time of sub-band APES (refer to Figure 11b) is just 0.84 h. Obviously, the two imaging results only have tiny differences, which are hardly recognized.

## 5. Conclusion

This paper has investigated the problem of suppressing spectral overlap in sub-band spectral estimation. The spectral overlap phenomenon is originated from the non-ideal behavior of the analysis filtering, *i.e.*, the filtering error. The error formation in convolution filtering was therefore discussed, based on which an extrapolation-based filtering strategy was proposed to greatly suppress spectral overlap. Several classical theories, including AR identification, Kalman prediction and the equiripple FIR filtering technique, are integrated into the strategy for linearly-optimal extrapolation. To resolve the “overfitting” in order determination with AIC and BIC, we modified the penalty terms for both criteria. The improved criteria adaptively adjust the penalty strength and avoid “overfitting” to some extent. Both 1D and 2D complex exponential signals are utilized to validate the performance of the proposed method. Moreover, we employed SAR image formation for a single-polarized SAR data, simulated based on electromagnetic theory, to testify the efficiency of our method. Future research will focus on developing more sophisticated methods for the problem of extrapolation, with which we can avoid model order determination and further improve the extrapolation precision.

## Figures and Tables

**Table 1.** Comparison of frequency estimation in full-band and sub-bands.

**Means and MSEs of Estimating Frequencies**	**Full-band**			**Sub-band**		

	**MUSIC**	**ESPRIT**	**SELF-SVD**	**MUSIC**	**ESPRIT**	**SELF-SVD**
*ω̄*_1_ (*ω*_1_ = –0.07500π)	–0.0750π	–0.0750π	–0.0790π	–0.0749π	–0.0749π	–0.0750π
*ω̄*_2_ (*ω*_2_ = –0.03125π)	–0.0309π	–0.0309π	∼	–0.0308π	–0.0313π	–0.0308π
*ω̄*_3_ (*ω*_3_ = 0.01250π)	0.0125π	0.0125π	0.0175π	0.0123π	0.0123π	0.0126π
*ω̄*_4_ (*ω*_4_ = 0.05625π)	0.0572π	0.0572π	∼	0.0555π	0.0557π	0.0555π
*ω̄*_5_ (*ω*_5_ = 0.10000π)	0.1000π	0.1000π	0.1027π	0.1000π	0.1000π	0.1000π
${\hat{\sigma }}_{{\bar{\omega }}_{1}}^{2}\times 10^{5}$	0.0072	0.0072	1.8126	0.0057	0.0058	0.0044
${\hat{\sigma }}_{{\bar{\omega }}_{2}}^{2}\times 10^{5}$	1.6462	1.6462	∼	0.1662	0.1259	0.1816
${\hat{\sigma }}_{{\bar{\omega }}_{3}}^{2}\times 10^{5}$	0.0125	0.0125	2.8847	0.0081	0.0074	0.0060
${\hat{\sigma }}_{{\bar{\omega }}_{4}}^{2}\times 10^{5}$	3.0443	3.0443	∼	0.1729	0.1199	0.1775
${\hat{\sigma }}_{{\bar{\omega }}_{5}}^{2}\times 10^{5}$	0.0360	0.0360	0.7379	0.0049	0.0041	0.0046

“∼” denotes meaningless estimates.

(Monte Carlo analysis: 100 runs). SELF-SVD, singular value decomposition-based method in a selected frequency band.

## Appendix

### A1. Residual Filtering Error Analysis

A stationary AR(*p*) process can be represented as:

(A1) $$x_{n}=\sum_{l=0}^{\infty }\alpha_{l}{ɛ}_{n-l}$$

where the coefficient series {α*_l_*} meets mean square convergence and can be calculated recursively [28]:

(A2) $$\{\begin{array}{l} \alpha_{l}=-\phi_{1}\alpha_{l-1}-\cdots -\phi_{p}\alpha_{l-p}=-\overset{\min\left(p,l\right)}{\underset{i=1}{\text{∑}}}\phi_{i}\alpha_{l-i}\\ \alpha_{0}=1,\alpha_{l}=0\left(l<0\right) \end{array}$$

Then *k*-step prediction formula in an alternative form is:

(A3) $${\hat{x}}_{n|n-k}=\overset{\infty }{\underset{l=k}{\text{∑}}}\alpha_{l}{ɛ}_{n-l}$$

The prediction error is $x_{n}-{\hat{x}}_{n|n-k}=\overset{k-1}{\underset{l=0}{\text{∑}}}\alpha_{l}{ɛ}_{n-l}$, and this mean that the variance and covariance are:

(A4) $$\text{E}\left\{x_{n}-{\hat{x}}_{n|n-k}\right\}=0$$

(A5) $$\text{E}\left\{{\left|x_{n}-{\hat{x}}_{n|n-k}\right|}^{2}\right\}=\sigma_{ɛ}^{2}\sum_{l=0}^{k-1}{\left|\alpha_{l}\right|}^{2}$$

(A6) $$\text{E}\left\{\left(x_{n_{1}}-{\hat{x}}_{n_{1}|n_{1}-k}\right){\left(x_{n_{2}}-{\hat{x}}_{n_{2}|n_{2}-k}\right)}^{*}\right\}=\{\begin{array}{cc} \sigma_{ɛ}^{2} & \underset{\left(l_{1},l_{2}\right)\in \Lambda }{\text{∑}}\alpha_{l_{1}}\alpha_{l_{2}}*\left|n_{1}-n_{2}\right|⩽k-1\\ 0 & \text{otherwise} \end{array}$$

respectively, where the set is:

$$\Lambda =\left\{\left(l_{1},l_{2}\right)|l_{1}-l_{2}=n_{1}-n_{2},0⩽l_{1},l_{2}⩽k-1\right\}$$

We define the desired output as *ỹ**_n_* and by analogy to Equation (11), we define its associated vector as:

(A7) $$\tilde{\text{y}}={\left[{ỹ}_{L_{G}},{ỹ}_{L_{G}+1,\cdots ,}{ỹ}_{L_{G}+N-1}\right]}_{\left(N\times 1\right)}^{\text{T}}$$

where:

(A8) $$ỹ=\tilde{\text{X}}\text{h}$$

and:

(A9) $$\tilde{X}={\left[\begin{array}{cccc} x_{L_{G}} & x_{L_{G}-1} & \cdots & x_{L_{G}-N_{o}}\\ x_{L_{G}+1} & x_{L_{G}} & \cdots & x_{L_{G}-N_{o}+1}\\ \vdots & \vdots & \ddots & \vdots \\ x_{L_{G}+N-1} & x_{L_{G}+N-2} & \cdots & x_{L_{G}+N-N_{o}-1} \end{array}\right]}_{\left(N\times \left(N_{f}\right)\right)}$$

All elements of **X̃** are truth samples, which are assumed to be known for derivation. Then, according to (A6), we can derive the MSE as:

(A10) $$\begin{array}{l} {\hat{\sigma }}^{2}=\frac{1}{N}\overset{N+L_{G}-1}{\underset{n=L_{G}}{\text{∑}}}\text{E}\left\{{\left|{ŷ}_{n}-{ŷ}_{n}\right|}^{2}\right\}\\ =\frac{1}{N}\overset{N_{o}-1}{\underset{n=L_{G}}{\text{∑}}}\overset{N_{o}}{\underset{m_{1}=n+1}{\text{∑}}}\overset{N_{o}}{\underset{m_{2}=n+1}{\text{∑}}}h_{m_{1}}h_{m_{2}}^{*}\text{E}\left\{\left({\hat{x}}_{n-m_{1}}-x_{n-m_{1}}\right)\left({\hat{x}}_{n-m_{2}}-x_{n-m_{2}}\right)*\right\}\\ +\frac{1}{N}\overset{N+L_{G}-1}{\underset{n=N}{\text{∑}}}\overset{n-N}{\underset{m_{1}=0}{\text{∑}}}\overset{n-N}{\underset{m_{2}=0}{\text{∑}}}h_{m_{1}}h_{m_{2}}^{*}\text{E}\left\{\left({\hat{x}}_{n-m_{1}}-x_{n-m_{1}}\right)\left({\hat{x}}_{n-m_{2}}-x_{n-m_{2}}\right)*\right\}\\ =\frac{\sigma_{ɛ}^{2}}{N}\overset{N_{o}-1}{\underset{n=L_{G}}{\text{∑}}}\underset{\left(m_{1},m_{2}\right)\in \Lambda_{1}}{\text{∑}}h_{m_{1}}h_{m_{2}}^{*}\underset{\left(l_{1},l_{2}\right)\in \Lambda^{\left(1\right)}}{\text{∑}}\alpha_{l_{1}}\alpha_{l_{2}}^{*}\\ +\frac{\sigma_{ɛ}^{2}}{N}\overset{N+L_{G}-1}{\underset{n=N}{\text{∑}}}\underset{\left(m_{1},m_{2}\right)\in \Lambda_{2}}{\text{∑}}h_{m_{1}}h_{m_{2}}^{*}\underset{\left(l_{1},l_{2}\right)\in \Lambda^{\left(2\right)}}{\text{∑}}\alpha_{l_{1}}\alpha_{l_{2}}^{*} \end{array}$$

where:

(A11) $$\begin{array}{c} \Lambda_{1}=\left\{\left(m_{1},m_{2}\right)|\begin{array}{l} \left|m_{1}-m_{2}\right|⩽k-1\\ n+1⩽m_{1},m_{2}⩽N_{o}\\ L_{G}⩽n⩽N_{o}-1 \end{array}\right\}\\ \Lambda^{\left(1\right)}=\left\{\left(l_{1},l_{2}\right)|\begin{array}{c} l_{1}-l_{2}=m_{2}-m_{1}\\ 0⩽l_{1},l_{2}⩽k-1\\ \left(m_{1},m_{2}\right)\in \Lambda_{1} \end{array}\right\}\\ \Lambda_{2}=\left\{\left(m_{1},m_{2}\right)|\begin{array}{l} \left|m_{1}-m_{2}\right|⩽k-1\\ 0⩽m_{1},m_{2}⩽n-N\\ N⩽n⩽N+L_{G}-1 \end{array}\right\}\\ \Lambda^{\left(2\right)}=\left\{\left(l_{1},l_{2}\right)|\begin{array}{c} l_{1}-l_{2}=m_{2}-m_{1}\\ 0⩽l_{1},l_{2}⩽k-1\\ \left(m_{1},m_{2}\right)\in \Lambda_{2} \end{array}\right\} \end{array}$$
